# Bridging the gap: a review of dose-investigation studies in paediatric investigation plans

**DOI:** 10.1186/1745-6215-14-S1-O42

**Published:** 2013-11-29

**Authors:** Lisa Hampson, Martin Posch, Julia Saperia, Anne Whitehead

**Affiliations:** 1 Lancaster University, Lancaster, UK; 2 Medical University of Vienna, Vienna, Austria; 3 Medicines and Healthcare Products Regulatory Agency, London, UK

## 

Recent regulatory changes place greater emphasis on ensuring that new medicinal products are appropriately licensed for use in children. The EU Paediatric Regulation stipulates that development in this group should follow a prospectively agreed paediatric investigation plan (PIP) outlining all of the studies to be conducted. For any medicine, finding the right dose is crucial if its benefits are to be properly evaluated. However, dose-finding in children may be challenging due to the variability of drug pharmacokinetics across age-groups and the limited sample sizes available. In such cases, uncertainty about likely drug effects in children may be reduced by extrapolating from existing data in relevant populations.

This review focuses on current strategies adopted in PIPs to support paediatric dose-recommendations. Data were extracted from 73 PIP opinions recently adopted by the Paediatric Committee of the European Medicines Agency. These PIP opinions represented a total of 79 development programs and comprised a total of 97 dose-investigation studies. This presentation discusses the key findings of this review. We highlight common assumptions made in extrapolations and find that few steps are often taken to validate needed assumptions. Sample sizes used to support dose-investigations are found to be highly variable across programs, with low numbers recruited from the very youngest age-groups.

